# Spatial and temporal patterns of bone formation in ectopically pre-fabricated, autologous cell-based engineered bone flaps in rabbits

**DOI:** 10.1111/j.1582-4934.2008.00137.x

**Published:** 2008-08-11

**Authors:** Oliver Scheufler, Dirk J Schaefer, Claude Jaquiery, Alessandra Braccini, David J Wendt, Jürg A Gasser, Raffaele Galli, Gerhard Pierer, Michael Heberer, Ivan Martin

**Affiliations:** a Departments of Surgery and of Research, University Hospital BaselBasel, Switzerland; b Novartis Institutes for Biomedical Research, Musculoskeletal DiseasesBasel, Switzerland

**Keywords:** bone tissue engineering, mesenchymal stem cells, vascularization, bone graft, bioreactor, flap pre-fabrication

## Abstract

Biological substitutes for autologous bone flaps could be generated by combining flap pre-fabrication and bone tissue engineering concepts. Here, we investigated the pattern of neotissue formation within large pre-fabricated engineered bone flaps in rabbits. Bone marrow stromal cells from 12 New Zealand White rabbits were expanded and uniformly seeded in porous hydroxyapatite scaffolds (tapered cylinders, 10–20 mm diameter, 30 mm height) using a perfusion bioreactor. Autologous cell-scaffold constructs were wrapped in a panniculus carnosus flap, covered by a semipermeable membrane and ectopically implanted. Histological analysis, substantiated by magnetic resonance imaging (MRI) and micro-computerized tomography scans, indicated three distinct zones: an outer one, including bone tissue; a middle zone, formed by fibrous connective tissue; and a central zone, essentially necrotic. The depths of connective tissue and of bone ingrowth were consistent at different construct diameters and significantly increased from respectively 3.1 ± 0.7 mm and 1.0 ± 0.4 mm at 8 weeks to 3.7± 0.6 mm and 1.4 ± 0.6 mm at 12 weeks. Bone formation was found at a maximum depth of 1.8 mm after 12 weeks. Our findings indicate the feasibility of ectopic pre-fabrication of large cell-based engineered bone flaps and prompt for the implementation of strategies to improve construct vascularization, in order to possibly accelerate bone formation towards the core of the grafts.

## Introduction

Vascularization plays a crucial supportive role during bone growth and fracture healing [1]. Indeed, formation and repair of bone can be prevented by angiogenesis inhibitors [2, 3], as well as stimulated by introducing vascular carriers [4]. Therefore, the first choice for surgical reconstruction of large bone defects (*e.g.* segmental defects wider than 6–8 cm [5] or large bone losses due to trauma, tumour and infection [6–8]) consists of autologous bone flaps, namely bone grafts with an internal vascular network supplied by large calibre vessels [5]. Using bone flaps, the healing process between the graft and host bone is reported to proceed not by creeping substitution, as in non-vascularized bone grafts, but similarly to fracture healing [9, 10], with union times as short as 3–5 months [11].

However, bone flaps often do not match the mechanical strength required at the bone defect, and thus may result in complications such as stress fractures in up to 25% of reconstructions, prolonging immobilization or leading to pseudarthrosis [10, 12]. Additional limits of autologous bone flaps include significant donor site morbidity [13–15], as well as complicated and time consuming shaping frequently required intraoperatively, due to the limited anatomically available sizes and shapes.

In order to overcome these limitations and to generate osteogenic grafts of pre-defined shapes, dimensions and mechanical properties, bone tissue engineering using various types of osteo-progenitor/stem cells in combination with appropriate carriers has been developed. Several studies have established that bone marrow stromal cells (BMSC), expanded in culture and loaded into porous ceramic scaffolds, are able to form bone tissue, both ectopically and orthotopically (see [16] for a recent review). Despite the report of few clinical cases [17], however, no convincing successes have been achieved in humans, most likely because of lack of sufficient vascular supply, resulting in immediate cell death after implantation [16, 18]. Indeed in small constructs, survival of the cells is supported by a short distance from the surrounding vasculature, while in constructs of clinically relevant size *(i.e.* a few cubic centimetres), the acceptable distance for diffusion of oxygen and nutrients is exceeded.

In order to solve the size limitations in bone tissue engineering, one possible option is to combine it with the concept of flap pre-fabrication, namely a two-stage surgical procedure whereby a new blood supply is transferred into a volume of tissue by the introduction of a vascular carrier [19–21]. Bone flap pre-fabrication is thus aimed at ectopically producing living bone tissue in pre-defined shapes and dimensions, which can then be transferred to a distant site with its blood supply [22]. Engineered bone flap pre-fabrication using a cell-based approach has been performed in a few animal models, using constructs of different sizes [23–25]. However the limiting dimension in these models never exceeded 6 mm, and thus would not be representative of a graft in a clinically relevant size. Moreover, the depths of vascularization and of bone tissue formation have not been assessed in any of these models, and therefore no indication on size limits in the pre-fabrication of engineered bone flaps is available.

In the present study, we first aimed at establishing a new model of ectopic pre-fabrication of autologous cell-based, large bone flaps in rabbits using porous ceramic scaffolds loaded with BMSC and wrapped by a panniculus carnosus flap as an external vascular carrier. We then used the model to investigate the spatial pattern and temporal progression of bone formation within the scaffolds. In order to exclude the possibility that such pattern was influenced by initially non-uniform cell distributions, we adapted and used a previously described direct perfusion bioreactor system for spatially homogenous cell seeding [26].

## Materials and methods

### Bone marrow harvest

The study was approved by the Animal Ethics Committee of the Swiss Federal Veterinary Office (http://bvet.admin.ch) and was conducted in accordance with the guidelines for the care and maintenance of animals at the University Hospital Basel. Bone marrow was harvested from 12 young adult New Zealand White (NZW) rabbits (Charles River Laboratories, Kisslegg, Germany) with an average body weight of 3.2 ± 0.4 kg. Animals were sedated by subcutaneous administration of 25 mg per kg of body weight ketamine hydrochloride (Ketaminol 5%® ad us. vet., Veterinaria AG, Züich, Switzerland) and 2.5 mg per kg of body weight xylazine (Narcoxyl 2%® ad us. vet., Veterinaria AG, Züich, Switzerland) before transfer to the operating room, where inhalation anaesthesia was initiated with isoflurane (Forene®, Abbott AG, Baar, Switzerland). Transcutaneous arterial oxygen saturation and heart rate were monitored with a pulse oximeter (NPB-290, Nellcor Puritan Bennett, Pleasanton, CA, USA) fixed to a front limb. Before surgery, single shot antibiotic prophylaxis with 15 mg per kg of body weight sulfadoxin-trimethoprim (Borgal® 24% ad us. vet Veterinaria AG, Züich, Switzerland) and 0.05 mg per kg of body weight buprenorphine (Temgesic®, Essex Chemie AG, Luzern, Switzerland) for pain relief were administered subcutaneously.

After the area over the iliac crests was shaved and prepped with povidone iodine solution (Betaseptic®, Mundipharma Medical Company, Basel, Switzerland), bone marrow was harvested by repeated puncture of both iliac bones (2–3 per side) using 20 gauge needles and 20 ml syringes filled with 1 ml of heparin sodium solution (Heparin-Na® 5’000 IU/ml, B. Braun Medical AG, Emmenbrüke, Switzerland), yielding average aspirate volumes of 17.3 ± 5.2 ml (range: 11.5- 27 ml) per animal. Fresh aspirates were diluted with a double volume of phosphate buffered saline (PBS, Gibco, Invitrogen Corporation, Basel, Switzerland), and centrifuged, resulting in the elimination of supernatant fat, blood clots, and small tissue particles. Nucleated cells were then stained with crystal violet 2.3% (Sigma-Aldrich, Fluka Chemie AG Buchs, Switzerland), counted and cultured as described below.

### BMSC expansion

The complete medium consisted of α-modified Eagle's medium (α-MEM with 4.5 mg/ml D-glucose and 0.1 mM non-essential amino acids, Gibco, Invitrogen Corporation, Basel, Switzerland) containing 10% foetal bovine serum (FBS), 1% penicillin-streptomycin-glutamate (PSG; 10’000 U/ml penicillin, 10,000 μg/ml streptomycin, and 200 mM glutamine), 1% HEPES buffer (1 M, Gibco, Invitrogen Corporation, Basel, Switzerland), and 1% sodium pyruvate (100 mM, Gibco, Invitrogen Corporation, Basel, Switzerland).

Nucleated cells from fresh aspirates were plated at a density of 1 **×** 10^5^ per cm^2^ in complete medium, further supplemented with 5 ng/ml FGF-2 (R&D Systems, Wiesbaden, Germany) and 10 nM dexamethasone (Sigma-Aldrich, Fluka Chemie AG, Buchs, Switzerland) to increase BMSC proliferation and osteogenic commitment [27]. All cell cultures were incubated in humidified atmosphere at 37°C / 5% CO2. Medium was first changed after 5 days, non-adherent cells were removed and fresh medium was added, with medium changes twice a week. When reaching sub-confluency, adherent cells were detached from the flasks using 0.3% collagenase (Worthington Biochemical Corporation, Lakewood, NJ, USA) and 0.05% trypsin/0.53 mM ethylenediaminetetraacetic acid (EDTA) (Gibco, Invitrogen Corporation, Basel, Switzerland), stained with trypan blue 0.4% (Sigma-Aldrich, Fluka Chemie AG, Buchs, Switzerland), counted and re-plated at a density of 3 **×** 10^3^ per cm^2^. When reaching again sub-confluency, BMSC were detached and used for all the experimental conditions described below.

### Clonogenicity and differentiation assays

Fibroblastic colony forming unit (CFU-f) assays were performed for each aspirate to determine the fraction of clonogenic BMSC. Briefly, 1 **×** 10^5^ nucleated cells were plated in duplicate in 28 cm^2^ tissue culture dishes and cultured as described above for a total of 14 days, such that colonies were clearly visible but not yet overlapping. Colonies were then rinsed with PBS, fixed with 4% buffered formaldehyde, stained with 1% methylene blue (Fluka Chemie GmbH, Buchs, Switzerland) and counted. Mean values of CFU-f frequency were used to derive the initial number of BMSC from each bone marrow sample, which was required to calculate the fold expansion and doubling time during the first passage.

Osteogenic differentiation assays were performed by plating in duplicate 3 **×** 10^3^ expanded BMSC per cm^2^ in complete medium, without (as control) or with further addition of 10 nM dexamethasone, 0.1 mM L-ascorbic acid-2-phosphate (Sigma-Aldrich, Fluka Chemie AG, Buchs, Switzerland) and 10 mM β-glycerolphosphate (Sigma-Aldrich, Fluka Chemie AG, Buchs, Switzerland) (osteogenic medium). After 3 weeks of culture, extracellular matrix mineralization and calcium nodule formation were assessed by staining with a 2% alizarin red solution (Sigma-Aldrich, Fluka Chemie AG, Buchs, Switzerland).

### Ceramic scaffolds

**Fig. 1:**
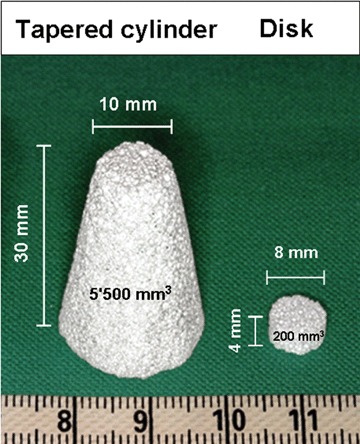
Porous ceramic scaffolds made of 100% hydroxyapatite (Engipore®) were fabricated in the shape of large tapered cylinders (30 mm height, 20 mm upper base diameter, 10 mm lower base diameter, 5.5 cm^3^ volume) or small disks (4 mm height, 8 mm diameter, 0.2 cm^3^ volume).

Porous ceramic scaffolds (porosity: 80 ± 3%, pore size distribution: 22%, <100 μm; 32%, 100–200 μm; 40%, 200–500 μm; 6%, **>** 500 μm) made of 100% hydroxyapatite, with a Ca/P ratio of 1.66 ± 0.5 (Engipore®, Fin-Ceramica, Faenza, Italy, http://www.fin-ceramicafaenza.com), were fabricated in the shape of large tapered cylinders (30 mm height, 20 mm upper base diameter, 10 mm lower base diameter, 5.5 cm^3^ volume) or small disks (4 mm height, 8 mm diameter, 0.2 cm^3^ volume) (Fig. 1). Scaffolds in the shape of tapered cylinders were employed to assess tissue ingrowth and new bone formation at different diameters, while disks were used to validate the *in vivo* osteogenic capacity of rabbit BMSC.

### Three-dimensional (3D) perfusion seeding

Expanded BMSC were seeded in disks or tapered cylinders using a previously described bioreactor system [26], based on the principle of direct perfusion of a single cell suspension through the interconnected pores of 3D scaffolds. Briefly, scaffolds were pre-wetted in complete medium and press-fitted into custom-made polycarbonate chambers (one scaffold per chamber), positioned at the bottom of two vertical Teflon-columns and connected with each other at their base through a U-shaped tubing. BMSC suspended in 10 ml of complete medium were introduced into the bioreactor and perfused through the ceramic pores in alternating directions at a flow rate of 1.2 ml/min for 18 hrs using a standard syringe pump (Programmable PHD 2000®, Harvard Apparatus, Holliston, MA, USA). A seeding density of 10 ×· 10^6^ cells per cm^3^ of ceramic was consistently used, corresponding to a total number of 55 **×** 10^6^ BMSC for tapered cylinders and 2 **×** 10^6^ BMSC for disks. All 3D perfusion cultures were incubated in humidified atmosphere at 37°C/5% CO_2_.

The uniformity of 3D perfusion seeding of rabbit BMSC in tapered cylinders was tested in four independent experiments. Following 18 hrs of perfusion as described above, cell-scaffold constructs were retrieved from the bioreactor, bisected longitudinally and cut into three cross-sections of 10 mm thickness. The uniformity of cell distribution within cross-sections was qualitatively assessed by staining with 3-(4,5-dimethylthiazol-2-yl)-2, 5-diphenyltetrazolium bromide (MTT) (Sigma-Aldrich, Fluka Chemie AG, Buchs, Switzerland).

### Construct implantation

**Fig. 2:**
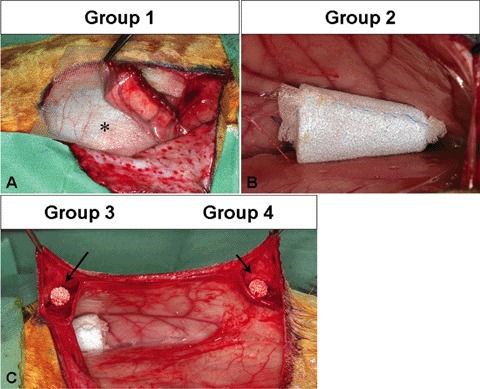
(**A**) In *group 1*, a large autologous cell-seeded tapered cylinder was first wrapped in a panniculus carnosus flap and then covered by a semi-permeable (Biobrane®) membrane. The flap wrapped around the cell-scaffold construct is depicted prior to its coverage with a sheet of Biobrane®, which is positioned under the flap (asterix). (**B**) In *group 2*, a large autologous cell-seeded tapered cylinder was first covered by a semipermeable membrane and then placed under the panniculus carnosus as a control. The cell-scaffold construct wrapped with Biobrane® is shown in situ under the panniculus carnosus. (**C**) In *groups 3* and *4*, two small disks were implanted subcutaneously, of which one was uniformly seeded with autologous BMSC and the other maintained cell-free. The cell-scaffold construct (left side, long arrow) and the empty scaffold (right side, short arrow) are shown *in situ* at opposite ends of the skin incision.

Cell-seeded ceramic scaffolds were retrieved from the bioreactor, rinsed in PBS, placed in sterile Falcon tubes pre-filled with PBS, and transferred to the operating room. After the surgical site on the dorsum of animals was shaved, disinfected and draped, a midline skin incision was made to expose the panniculus carnosus. An anteriorly pedicled 6.5 cm wide and 8 cm long panniculus carnosus flap centred over its axial vascular pedicle was raised on one side, whereas a sub-muscular pocket was created on the other side. Then, two large autologous cell-scaffold constructs were implanted on opposite sides: one was first wrapped in a panniculus carnosus flap and the flap then covered by a semipermeable membrane (*group 1*, vascularized condition, Fig. 2A), whereas the other one was first covered by a semipermeable membrane and then placed under the pan-niculus carnosus as a control (*group 2*, non-vascularized condition, Fig. 2B). Instead of silicone sheeting, which has previously been used to prevent vascular invasion of pre-fabricated flaps from surrounding tissues [25], a semipermeable membrane (Biobrane®, UDL Laboratories Inc., Rockford, IL, USA) was employed in the present study. The material is permeable to oxygen and nutrients, and allows drainage of exudate through small pores, thereby preventing the accumulation of wound fluid and seroma formation. The membrane, which is usually applied on the skin as a temporary substitute, consisted of a silicone surface, which was applied in contact with the cell-scaffold construct, and a nylon-collagen surface, facing the surrounding soft tissues. The membrane edges were meticulously sutured with non-resorbable synthetic monofilament materials (Prolene® 4.0, Ethicon Inc., Norderstedt, Germany) to prevent construct invasion by surrounding tissues. The control group (*group 2*) was aimed at testing potential adverse reactions of Biobrane® (*e.g.* immunological response to the collagen surface), as well as to establish the baseline tissue formation, in the presence of oxygen and nutrient diffusion but in the absence of vascular invasion. In addition, two small disk-shaped scaffolds were implanted subcutaneously at opposite ends of the skin incision in the same animal (Fig. 2C): one was uniformly seeded in the perfusion system with autologous BMSC to determine their ectopic osteogenic capacity (*group 3*), while the other one was maintained cell-free, as a control to investigate spontaneous tissue formation within the ceramic material *(group 4).*

### Construct explantation

Rabbits were euthanized by intravenous injection of pentobarbital (Nembutal®, Sanofi, Basel, Switzerland) after 8 or 12 weeks of implantation (n = 6 animals per time point, randomly assigned), and constructs retrieved. Engineered bone flaps *(group 1)* were harvested together with the surrounding semipermeable membrane; the vascular pedicle was always isolated at the flap base and inspected for patency. After division of the vascular pedicle, the membrane and the flap were removed, the construct was exposed and assessed macroscopically for signs of infection and external vascularization. Non-vascularized constructs *(group 2)* were also harvested together with the membrane and assessed macroscopically for signs of local wound reaction, infection and vascular penetration through the membrane pores with untoward vascularization of the constructs. Samples were then assessed for magnetic resonance imaging (MRI) and micro-computerized tomography, and finally processed for histological analyses.

### Histology

All explanted constructs were bisected along the vertical axis; one-half was fixed in 4% formalin supplemented with 1% calcium chloride and embedded in polymethylmethacrylate, while the second was fixed in 4% buffered formaldehyde, decalcified (Osteodec®, Bio-Optica, Milan, Italy) and embedded in paraffin. For tapered cylinders, the two-halves were histologically assessed at three different levels, by cross-sectioning at the levels of 18 mm (level A), 15 mm (level B) and 12 mm (level C) of cylinder diameter. Undecalcified samples were histologically assessed by 170 ± 3 μm-thick sections stained with McNeal tetrachrome, in order to quantify the extent of connective and bone tissue ingrowth as described below, while decalcified specimens were assessed by 5 μm-thick sections stained with haematoxylin/eosin, in order to quantify the amount and uniformity of bone tissue formation, as described below.

### Quantitative histomorphometry

Connective and bone tissue ingrowth was assessed by computer-assisted analysis of digitized microscopical images of McNeal tetrachrome stained sections. Tissue ingrowth was measured using a digital calliper as the distance from the outer border of the scaffold to the inner limit of tissue formation perpendicular to the circumference of the scaffold. For each disk and for each diameter in tapered cylinders, three sections were analysed and three measurements per section were performed.

The amount and uniformity of bone formation were assessed by computer-assisted histomorphometry of microscopical images acquired both in transmitted and fluorescent light (excitation wavelength 546 nm, emission wavelength 590 nm) from haematoxylin/eosin stained sections, as previously described [28]. Briefly, the amount of bone tissue was quantified in each image as the area of fluorescent tissue, and the area available for tissue ingrowth *(i.e.* pore space) was determined by subtracting the area of undegraded scaffold, quantified in the transmitted light images, from the total cross-sectional area. For each image, the amount of bone tissue was then calculated as a percentage of the total available pore space. For each disk and for each diameter in tapered cylinders, a total of nine sections were analysed by acquisition of six images per section, in order to cover most of the total cross-sectional area. The amount of bone formation was calculated as the average and standard deviation of the percentages measured in the nine sections.

### Magnetic resonance imaging (MRI)

High resolution MRI was performed on a 3.0 Tesla head scanner (Magnetom Allegra™, Siemens Medical Solutions, Erlangen, Germany) using the standard head coil for signal excitation and reception. A 3D turbo-spinecho sequence with the following parameters was used for imaging: 2000 ms repetition time (TR), 15 ms echo time (TE), 384 **×** 144 **×** 86 matrix size, 154 **×** 77 **×** 58 mm^3^ field-of-view (FOV), five turbo-factor. To increase the signal-to-noise ratio, eight signal averages were acquired, resulting in a total measurement time of approximately 12 hrs. To optimize the T1 contrast between ingrowing tissue and porous ceramic, an inversion recovery magnetization preparation with an inversion time (TI) of 400 ms was applied. Images were Fourier-interpolated to a voxel size of 0.4 **×** 0.5 **×** 0.4 mm^3^. In all vascularized constructs *(group 1),* the region filled with connective tissue was distinguished from the empty pores by a segmentation procedure, thus allowing to quantify the distance of tissue ingrowth from the construct edges. The accuracy of MRI in detecting connective tissue ingrowth was validated by comparing measurements with those derived from analysis of histological sections in corresponding regions *(i.e.* at level B).

### Micro-computed tomography (μCT)

A high-speed μCT scanner for *in vivo* applications (vivaCT 40™, Scanco Medical AG, Bassersdorf, Switzerland) with a maximum scan diameter of 20–38 mm and a maximum scan length of 145 mm at a nominal isotropic resolution of 10–72 μm was used. As volume of interest (VOI), cross-sections of 1.5 mm thickness at 15 mm diameter of vascularized *(group 1)* and non-vascularized *(group 2)* tapered cylinders, corresponding to the level B of histological specimens, were scanned at a resolution of 20 μm. 2D images acquired by the system from a total of 88 slices in the VOI were used to re-construct 3D images of the outer 2 mm of tapered cylinders, previously determined by histology as the area where bone tissue formation was limited. While a clear distinction between ceramic and newly formed bone could not be established based on the scans, the technique was used to quantify the combined amount of ceramic plus newly formed bone in each construct, excluding the remaining soft tissues. The threshold employed for the segmentation was set at 325 mg/cm^3^ based on an initial validation of the method, using ceramic specimens where the depth of bone formation was histologically established.

### Statistics

Statistical calculations were performed using the SPSS 15.0 statistical software package (SPSS Schweiz AG, Züich, Schweiz). All data from the *in vitro* studies on cell characterization and seeding efficiency/uniformity are presented as mean **±** standard deviation of values determined for each bone marrow aspirate and each construct. Differences between groups were assessed using Mann–Whitney *U* -tests and, unless otherwise specified, were considered statistically significant with *P***<** 0.05.

## Results

### BMSC cultures

The total number of nucleated cells harvested from processed bone marrow aspirates averaged 124.8 **×** 10^6^**±** 20.3 **×** 10^6^ (7.8 **×** 10^6^**±** 2.4 **×** 10^6^ cells per ml of aspirate), of which 0.018 ± 0.012% were fibroblastic clonogenic cells (CFU-f). The average proliferation rate of BMSC was 0.78 ± 0.13 doublings per day during the first passage and 0.68 ± 0.01 doublings per day during the second passage. The CFU-f percent and proliferation rate of rabbit BMSC were comparable to those previously reported for other mammalian species, including human [29] and sheep [30]. Deposition of mineralized extracellular matrix, in the form of nodules positively stained by alizarin red, was evident using cells of eight out of 12 animals following culture with osteogenic medium, and not detectable following culture with control medium.

Preliminary experiments on perfusion seeding of the tapered cylinders indicated a uniform spatial distribution of seeded cells within cross-sections at different diameters, as qualitatively assessed by MTT stain (data not shown).

### Explant analyses

**Fig. 3:**
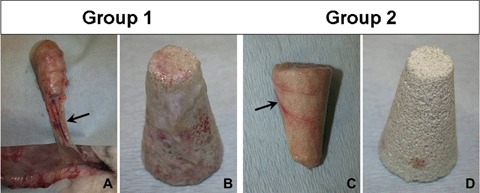
(**A**) In group 1, the intact vascular pedicle (arrow) is demonstrated after dissection of the pre-fabricated flap from the surrounding tissues. (**B**) After removal of the flap, invasion of the outer pores by the flap tissue is visible macroscopically over the entire construct surface. (**C**) In group 2, the outer nylon-collagen surface of Biobrane® shows vascular invasion (arrow) from the surrounding tissues. (**D**) However, after removal of the membrane, no tissue or vessels have penetrated the membrane and grown into the construct pores.

Macroscopically, all implants were intact and could be further processed as planned, with the exception of one (from *group 2)* which was infected and was thus excluded from the study. Tapered cylinders implanted under vascularized conditions *(group 1)* could be dissected from surrounding tissues and maintained an intact vascular pedicle (Fig. 3).

**Fig. 4:**
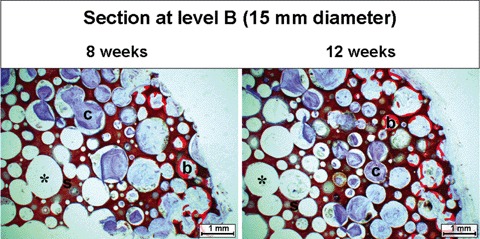
Representative histological sections of explanted tapered cylinders from *group 1* stained with McNeal tetrachrome after 8 or 12 weeks of implantation. Constructs displayed three distinct zones: an outer one, including newly formed bone tissue (**b**, red stain); a middle zone, formed by a fibrous connective tissue (**c**, blue stain); and a central zone, containing only empty pores (*). The undegraded scaffold (s) is stained brown.

**Fig. 5:**
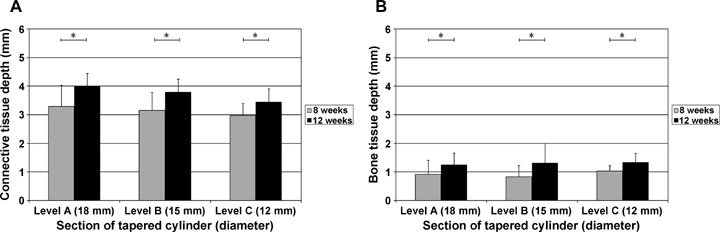
In explanted tapered cylinders from group 1, the depth of connective tissue (**A**) and bone tissue (**B**) ingrowth, as assessed by histology, was consistent in cross-sections at different diameters and significantly higher after 12 weeks as compared to 8 weeks of implantation (*=*P*<0.05).

Histological assessment indicated absence of vasculature or soft tissue ingrowth into the tapered cylinders in the presence of the semipermeable membrane *(group 2).* Instead, vascularized connective tissue was found throughout the entire available pore spaces in disks (*groups 3* and *4)* and in the outer pore spaces in tapered cylinders from *group 1,* leaving a central necrotic core with empty pores (Fig. 4). In constructs from *group 1,* the depth of connective tissue ingrowth was consistent in cross-sections at different diameters and was significantly higher after 12 weeks (3.7 ± 0.6 mm) as compared to 8 weeks of implantation (3.1 ± 0.7 mm) (Fig. 5A).

**Fig. 6:**
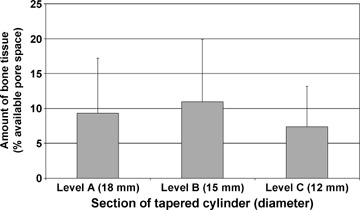
In explanted tapered cylinders from group 1, the amount of bone tissue, as assessed by quantitative histomorphology of the outer 2 mm of histological cross-sections, was similar at different diameters.

Bone tissue was not detected under non-vascularized conditions *(group 2)* or in cell-free disks *(group 4),* whereas it was formed in nine out of 12 animals within autologous cell-based constructs implanted under vascularized conditions *(groups 1* and *3*). Interestingly, except for one case, we observed a direct correspondence between the capacity of cells from different animals to form bone tissue *in vivo* and to deposit mineralized matrix *in vitro.* In cell-loaded disks *(group 3*) newly formed bone tissue was distributed irregularly, though predominantly in the outer pores, whereas in tapered cylinders *(group 1)* it was clearly confined within an outer region (Fig. 4). The depth of bone tissue formation in constructs from *group 1* was consistent in cross-sections at different diameters and was significantly higher after 12 weeks (maximum depth: 1.8 mm; average: 1.4 ± 0.6 mm) as compared to 8 weeks of implantation (maximum depth: 1.3 mm; average: 1.0 ± 0.4 mm) (Fig. 5B). Bone matrix filled an average of 6.6 ± 9.8% and 9.1 ±7.6% of available pore space respectively in disks *(group 3*) and in the outer 2 mm of tapered cylinders *(group 1),* with comparable amounts in cross-sections at different diameters (Fig. 6).

**Fig. 7:**
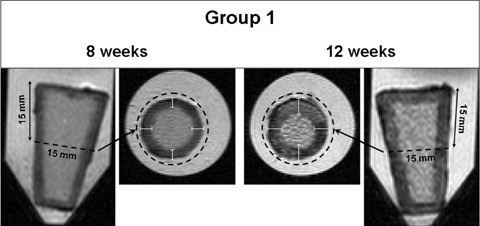
Magnetic resonance images of explanted tapered cylinders from group 1 demonstrate spatial differences in the T1 signal, allowing to distinguish between an outer (dark grey) zone, corresponding to the region filled with connective tissue, and an inner (light grey) zone, where pores were essentially empty. At 15 mm diameter of tapered cylinders, the depth of the outer zone was higher after 12 weeks as compared to 8 weeks of implantation.

MRI of explanted cylinders indicated clear spatial differences in the T1 signal of constructs under vascularized conditions *(group 1),* allowing to distinguish between an outer and an inner zone (Fig. 7). The depth of the outer zone, quantified at a 15-mm diameter cross-section of the cylinders (level B), was significantly higher after 12 weeks (4.0 ± 0.6 mm) as compared to 8 weeks of implantation (3.5 ± 0.7 mm), and corresponded to the histologically assessed region filled with connective tissue. The signal intensity measured in the inner zone was similar to that measured throughout cylinders under non-vascularized conditions *(group 2),* and thus corresponded to the absence of tissue in the necrotic core (data not shown).

**Fig. 8:**
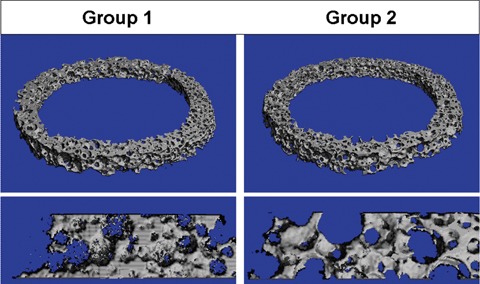
Micro-computerized tomography scans of the outer 2 mm of explanted tapered cylinders implanted under vascularized (*group 1*) or non-vascularized conditions (*group 2*). Images were segmented at a density threshold at 325 mg/cm^3^, allowing to distinguish the combination of bone tissue and ceramic scaffold (filled volume) from the connective tissue (void volume).

Micro-computerized tomography scans of explanted cylinders allowed to distinguish between the connective tissue and the combination of bone tissue and ceramic scaffold, based on a density threshold. As compared to control cylinders under non-vascularized conditions *(group 2),* deposition of new bone in the pores of tapered cylinders implanted under vascularized conditions *(group 1)* was characterized by changes in all morphometric parameters (Fig. 8 and Table 1). In particular, constructs from *group 1* had a significantly higher trabecular number (1.31-fold), tra-becular volume (1.25-fold) and trabecular thickness (1.09-fold), lower trabecular separation (1.43-fold), higher connectivity density *(i.e.* the number of trabecular connections in a given volume; 1.57-fold), higher structure model index *(i.e.* ratio of convex to concave structures; 2.03-fold) and higher density of the mineralized phase (1.26-fold). Differences between *group 1* and *group 2* in all trabecular characteristics, with the exception of the trabecular thickness, were more marked after 12 weeks as compared to 8 weeks of implantation, although these trends were not statistically significant.

**Table 1:** Micro-computerized tomography parameter†

	*Group 1* Vascularized condition	*Group 2* Non-vascularized condition
Trabecular number (n)	6.98 ± 0.85**	5.32 ± 0.99
Trabecular volume (%)	42.27 ± 4.11**	33.84 ± 4.03
Trabecular thickness (mm)	0.14 ± 0.01**	0.13 ± 0.01
Trabecular separation (mm)	0.16 ± 0.03**	0.23 ± 0.05
Connectivity density (1/mm^3^)	124.61 ±37.17**	79.36 ± 36.00
Structure model index‡	1.54 ± 0.52**	0.76 ± 0.015
Material density (mg/cm^3^)	595.10 ± 41.15**	472.79 ± 44.36

† The typical histomorphometric parameter is here referring to the combined amount of ceramic plus newly formed bone.

‡ The index reflects the ratio of convex to concave structures and is increased if bone tissue is deposited on the concave ceramic pore surfaces.

** Statistically significant difference between vascularized and non-vascularized conditions (P<0.01).

## Discussion

In this study, we first established a new rabbit model of ectopic pre-fabrication of autologous BMSC-based engineered bone flaps, in a size which is sufficiently large to detect critical size-related limits in tissue ingrowth and bone formation. We then used the model to establish that bone formation is initially limited to the outer periphery of the constructs and progressively proceeds towards the centre, preceded by connective tissue formation and reaching a maximum depth of 1.8 mm after 12 weeks.

The developed model was based on the following main components: (*i*) the panniculus carnosus flap, which was sufficiently extended to entirely cover the large cell-scaffold constructs and which included an abundant vascular network supplied by defined axial vessels and a vascular pedicle [31]; (*ii*) the semipermeable membrane, which was wrapped around the pre-fabricated bone flap in order to prevent adherence to the surrounding tissue and thus potentially allow orthotopic transfer through the vascular pedicle at a later stage [25]; (*iii*) the porous ceramic material, previously used in conjunction with BMSC of various species to generate osteogenic grafts [29, 30] and here shaped in the form of tapered cylinders to establish that the process of bone tissue formation in the scaffold is effectively regulated by the distance from the flap; (*iv*) the perfusion bioreactor system, which was instrumental to achieve a uniform loading of the cells into the large scaffold, and thus to exclude that the pattern of bone tissue formation was possibly related to that of initial cell distribution [26].

Histological analyses of connective tissue and bone tissue ingrowth in the construct were complemented by non-invasive techniques allowing 3D rendering of the data, namely MRI and micro-computerized tomography scans. We first verified that MRI allowed to distinguish between pores remaining empty and pores filled with tissue, based on histological assessments performed in corresponding regions. We then used the method to establish that tissue ingrowth from any side of the construct was dependent upon the distance from the flap and proceeding towards the centre at the rate of about 55 μm per day during the first 8 weeks, and of about 21 μm per day between 8 and 12 weeks. The relatively lower rate, as compared to that of at least 150 μm per day reported for vascular invasion of orthotopical cancellous or cortical bone grafts in rabbits [32], could be due to (*i*) consequences of tissue injury during flap pre-fabrication, since the flap was used immediately after harvesting, with no ischaemic pre-conditioning, and/or (*ii*) the composition, structure and porosity of the different graft materials.

Micro-computerized tomography scans in the outer 2 mm of vascularized constructs indicated significant changes in most of the assessed morphometric parameters as compared to the non-vas-cularized grafts containing no bone tissue. This finding confirms that micro-computerized tomography parameters, previously shown to correlate with histomorphometrical bone measurements [33, 34], may also be used to characterize newly formed bone tissue within porous scaffolds. The fact that the technique was not able to distinguish between the ceramic scaffold and newly formed bone tissue highlights that more sophisticated approaches (*e.g.* those based on synchrotron radiation) are necessary to replace conventional histomorphometry in bone tissue engineering [35].

Histological analysis of tissue formation within cross-sections of the pre-fabricated flap indicated three distinct zones: an outer one, including newly formed bone tissue; a middle zone, formed by a fibrous connective tissue and a central zone, containing only remnants of cells and essentially necrotic. Between 8 and 12 weeks, the region containing bone tissue extended towards the centre of about 0.5 mm, corresponding to a rate of about 18 μm per day. The rate is relatively lower as compared to that recently reported for engineered bone tissue in goats (about 100 μm per day), probably due to the fact that in that study the implantation was orthotopic and the ceramic scaffolds intrinsically osteoinduc-tive [36]. In our model, the use of unlabelled autologous cells did not allow to determine whether survival of the implanted cells was limited to a specific region, nor whether bone formation between 8 and 12 weeks was based on osteoconduction from the initially formed bone or on activation of implanted cells which remained latent in the first period after implantation. However, the clear centripetal temporal progression found indicates that the process of bone formation was not relying on the diffusion of nutrients from the construct edge, which would be expected to remain similar over time or decrease as new matrix is formed, but rather on the progressive vascularization of the graft.

The findings of the present study thus prompt for the implementation of strategies to improve construct vascularization, in order to possibly accelerate bone formation towards the core of the grafts. In this context, several promising approaches have been outlined, although to our knowledge not yet successfully validated in the context of bone tissue engineering. One of these options is based on cell seeding of a construct supplied by an arteriovenous loop only a few days after its generation, during the period of intense angiogenesis [37]. Alternatively, specific factors supporting recruitment and stabilization of blood vessels, including vascular endothelial growth factor (VEGF) and platelet derived growth factor (PDGF), could be delivered from the construct, possibly using vehicles allowing defined release kinetics [38] or genetic cell modifications avoiding aberrant haemangioma [39]. Yet another strategy would be based on the co-seeding of endothelial cells, which would form a network capable to rapidly connect with the host vasculature in a process called ‘inosculation’[40]. Towards the latter possibility, the use of adipose tissue-derived cells would allow the isolation of both osteoblastic- and endothelial-lineage cells from the same source, and thus the engineering of both osteogenic and vasculogenic grafts [41]. Last but not least, it should be considered that the properties of the scaffold used as cell carrier play an important role to regulate graft vascularization. Thus, future investigations will have to further elucidate the effect of scaffold chemistry (*e.g.* composition, degradation rate) and structure (*e.g.* porosity, pore interconnectivity, interconnected pore size) on angiogenic processes [42, 43].

## Conclusions

In this study, we developed a model of ectopic pre-fabrication of large engineered bone flaps in rabbits, by combining the concepts of cell-based bone tissue engineering and of flap pre-fabrication. Analysis of bone formation demonstrated the viability of the developed concept and established size-related limits in bone flap pre-fabrication in the model. In parallel to the implementation of strategies to improve construct vas-cularization, it will now be important to assess whether orthotopic transfer of the ectopically pre-fabricated engineered bone flap would lead to a superior efficacy in the treatment of critically sized bone defects, as compared to direct orthotopic implantation of the grafts.
